# Nutritional status associated with clinical outcomes in children with solid tumors: A retrospective cohort study from China

**DOI:** 10.1002/cam4.6798

**Published:** 2023-12-18

**Authors:** Yongzhen Li, Zhongying Lu, Ao Ma, Wei Yao, Rui Dong, Kai Li, Min Wu, Kuiran Dong, Tian Qian

**Affiliations:** ^1^ Clinical nutrition Department Children's Hospital of Fudan University Shanghai China; ^2^ Child Health Management Centre Starkids Children's Hospital Shanghai China; ^3^ Pediatric Clinical Research Unit, Department of Research Management Children's Hospital of Fudan University Shanghai China; ^4^ Department of Oncology Children's Hospital of Fudan University Shanghai China

**Keywords:** children, clinical outcomes, malnutrition, nutritional status, solid tumors, undernutrition

## Abstract

**Objective:**

To investigate the long‐term changes in nutritional status in children with solid tumors during treatment and the relationship between nutritional status and clinical outcomes.

**Methods:**

This study was a retrospective medical records review of data from children who were diagnosed with solid tumors and followed up for more than 3 months from January 2016 to December 2021 in China. Patient demographics and clinical information, including nutritional status, parenteral nutrition use, intensive care unit (ICU) transfers, infection during hospitalization, hospitalization frequency, length of stay, hospitalization costs and antibiotic costs, were collected to analyze the nutritional status of children with different types of solid tumors, the dynamic changes in nutritional status during treatment, and the relationship between nutritional status and clinical outcomes.

**Results:**

Among the 764 patients (383 males (50.1%); 381 females (49.9%); mean age: 2.58 years), 41.6% of the solid tumors were neuroblastomas, 17.1% were hepatoblastomas, and Wilms tumors as the third most common solid tumors (8.9%). The median follow‐up duration was 6 months (range: 3–40 months). At diagnosis, the proportion of children who were undernourished (underweight and wasting) versus overweight or obese were 26.71% versus 5.21% (25.86% vs. 2.89% in the third month; 29.77% vs. 2.28% in the sixth month; 24.77% vs. 3.27% in the 12th month). The body mass index *Z* scores decreased from the initial values after the first month (−0.56 (−1.47, 0.23) vs. −0.44 (−1.29, 0.41)) but improved later and decreased again at 6 months. The children in the undernutrition group had longer hospital stays (*p* < 0.001), higher hospitalization costs (*p* < 0.001), higher antibiotic costs (*p* < 0.001), a higher risk of neutropenia (OR = 4.781 (95% CI: 1.571–14.553), *p* = 0.006), and a higher risk of ICU transfers (OR = 1.498 (95% CI: 1.010–2.224), *p* = 0.044). No significant differences in those associations by malnutrition and infection, ICU duration, or length of parenteral nutrition were observed.

**Conclusion:**

There is a considerable prevalence of malnutrition in children with solid tumors. Malnutrition is related to adverse clinical outcomes and increases in total hospital expenses and antibiotic costs.

## 1 INTRODUCTION

Cancer is the second leading cause of death in children aged 1–14, and unlike adults, the most common types of cancer in children are leukemia, brain and spinal cord tumors and various solid tumors, such as neuroblastomas, Wilms tumors, lymphomas, and rhabdomyosarcomas.^1^ Reports show that 85% of children with cancer survive 5 years or more, which indicates that there has been a very large increase in survival time since the mid‐1970s due to advanced and intensive therapies and treatment advances in recent decades.^1^ However, the tumors themselves and related therapies still produce a series of adverse rapid and delayed effects on patients,^2^, ^3^ so it is very important to improve the treatment standard and reduce the occurrence of adverse events during and after treatment.

Malnutrition refers to inadequate, excessive or unbalanced intake of energy or nutrients and can produce overnutrition, undernutrition, which includes stunting, wasting and underweight, and micronutrient‐related malnutrition.^4^ The prevalence of undernutrition varies considerably according to different studies, with reports indicating 6%–50% in children with solid tumors,^5^, ^6^, ^7^, ^8^, ^9^ and 50% of patients with neuroblastoma are diagnosed with malnutrition.^10^ Weight loss and malnutrition are often due to metabolic changes caused by tumors, adverse effects on the digestive system (nausea, vomiting, loss of appetite) caused by drugs and other therapies during treatment,^10^ and adverse psychological factors in patients.^11^, ^12^, ^13^ Malnutrition can further affect treatment tolerance, which may impact drug distribution and metabolism, so dose adjustments must be considered to prevent an increased incidence and severity of toxicity.^14^ Patients may be unable to receive further treatment, or they may stop or give up treatment due to poor nutritional status. For survivors, nutritional status also affects quality of life and outcomes,^15^, ^16^ resulting in a vicious cycle.

Awareness is growing regarding nutritional support interventions for patients to maintain proper nutritional status, leading to short‐term and long‐term benefits. Gallon et al. conducted two 3‐year cohort studies in pediatric patients with solid tumors to determine the effect of nutritional support on disease. The results showed that in the period of intensified nutrition therapy, the need for antimycotic therapy was significantly reduced, the time from diagnosis to completion of treatment was significantly shortened, and the total percentage of surviving children increased (75% vs. 60.3%).^17^

Malnutrition is an urgent problem in children with solid tumors. To date, studies on the nutritional status of patients with pediatric solid tumors have been mostly cross‐sectional studies, and there are few studies on the long‐term trends of nutritional status and its relationship with clinical outcomes, especially in China. In light of this, we performed a study that was designed to (a) evaluate the nutritional status of children with different types of solid tumors at diagnosis; (b) explore the trend in nutritional status during treatment; (c) analyze the relationship between nutritional status and clinical outcomes; and (d) provide scientific evidence for the formulation of accurate and effective nutritional support practices for children with solid tumors.

## 2 MATERIALS AND METHODS

### 2.1 Study design and participants

A retrospective cohort study was performed in the oncology department at Children's Hospital of Fudan University. The eligibility criteria included admission to the Department of Oncology for the first time from January 2016 to December 2021; solid tumor diagnosis confirmed by pathology and clinical indicators; age 0–18 years old; and available information on demographics, nutritional status and clinical outcomes of the patients with follow‐up greater than 3 months. The exclusion criteria were palliative intent treatment, the absence of solid tumors, diagnosis before January 2016, readmission follow‐up less than 3 months, and lack of height, weight and biochemical examination data.

A total of 2006 patients were diagnosed with solid tumors between 2016 and 2021. Patients with only one admission record who were not followed up in our hospital for reasons such as hospital transfer or treatment abandonment (152, 7.5%), those who were admitted before 2016 and readmitted for chemotherapy or surgery (1050, 52.3%), and patients with missing anthropological measurement data (40, 2.0%) were excluded, resulting in the enrollment of 764 children in the study (Figure S1). Ethical approval was obtained from the Medical Ethics Committee of the Children's Hospital of Fudan University (number: IRB (2021)558).

### 2.2 Procedure

Children who received conventional treatment during the period were continuously enrolled in the cohort. The children's growth and clinical information were measured at baseline and monthly thereafter. The nutritional status of the children was grouped at baseline and at each readmission. Body mass index (BMI) was calculated as weight (kg)/height^2^ (m^2^). Height for age (H/A), weight for age (W/A), and BMI Z values were calculated using an online *Z* value calculator that has been validated by the World Health Organization (WHO). Patients were classified into two categories depending on their BMI Z values. Undernutrition was considered for patients with values below the (−)1 standard deviation (SD) *Z* score, while the control group included those patients whose values were over the (−)1 SD *Z* score, either for H/A (stunted) or BMI for age (BMI/A)(wasted), in all age groups. Undernutrition patients were further classified into mild (−1 ≥ *Z* score > −2), moderate (MAM, −2 ≥ *Z* score > −3) and severe acute malnutrition (SAM, *Z* score ≤ −3).^18^ Overweight and obese children and adolescents were classified if they had BMI Z values greater than +2 and + 3 SD z scores, respectively. Weight measurements were collected weekly during hospitalization.^18^, ^19^

### 2.3 Demographics and clinical parameters

Demographic data (age, sex, nationality), nutritional status (height, weight, total protein, albumin, prealbumin, retinol‐binding protein), and clinical data (clinical diagnosis, pathological diagnosis, laboratory data, intensive care unit (ICU) transfers, infection during hospitalization, neoplasm staging (to determine the severity of the disease), tumor metastasis, parenteral nutrition (PN) implementation, outcomes, surgery, hospitalization costs and antibiotic costs) were obtained from the electronic medical records.

The diagnosis of infection during hospitalization was based on the patient's clinical symptoms (body temperature, heat peak duration) and laboratory results (white blood cell count, level of serum procalcitonin (PCT), blood culture results, C‐reactive protein (CRP), absolute neutrophil count (ANC), neutrophil percentage).^20^ Neutropenia was defined as an ANC of <500 cells/mm^3^ or an ANC expected to decrease to <500 cells/mm^3^ during the next 48 h.^21^, ^22^ Body weight was measured using a calibrated electronic scale (accuracy 0.01 kg, for infants to the nearest 0.001 kg). Height was measured using a calibrated height meter (accuracy 0.1 cm).

### 2.4 Data analysis and statistics

Data analyses were performed with SPSS 25.0 (IBM SPSS Statistics for Windows, Version 25.0. IBM). Patient demographics and clinical characteristics are presented as the median (P_25_, P_75_) and mean ± SD for continuous variables, and categorical and nominal variables are presented as frequencies (percentages). Differences between patients with malnutrition and the control group were tested with Fisher's exact test and the Chi‐squared test for categorical variables. The two‐sample *t* test and Mann–Whitney *U* test were used to describe variable characteristics and identify the relationship between continuous variables in the different groups. Multilevel analyses were performed to determine the effects of nutritional status on clinical outcomes. The regression models were fitted to examine the association by adjusting for age, sex, diagnosis, disease severity, tumor metastasis, and surgery. All variables were evaluated for multicollinearity. Risk was calculated as the odds ratio (OR) and 95% confidence interval (CI), and binary logistic regression was used for the multivariate analysis. A two‐sided *p* value level of 0.05 was considered statistically significant.

## 3 RESULTS

### 3.1 Patient demographics and clinical characteristics

**FIGURE 1:**
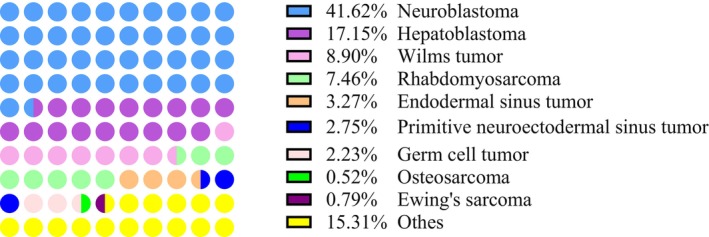
Proportion and distribution in all subset types of solid tumor.

The study included 764 patients (males/females = 383/381) with a median age of 2.58 years (1.21–4.66, range: 0.08–16.58 years). The median follow‐up duration was 6 months (range: 3–40 months). The most common solid tumors were neuroblastoma (41.6%) and hepatoblastoma (17.1%). Wilms tumor was the third most common solid tumor (8.9%), followed by rhabdomyosarcoma (7.5%) and endoembryonic sinus tumor (3.3%) (Figure 1). Table 1 shows the demographics and clinical characteristics of the patients.

**TABLE 1:** Demographics and clinical characteristics of patients.

	Value (*N*)	Percentage (%)
Age (years)	2.58 (1.21,4.66)	
Sex (*n*, %)		
Male	383	50.1
Female	381	49.9
Types of solid tumors		
Neuroblastoma	318	41.6
Hepatoblastoma	131	17.1
Wilms tumor	68	8.9
Rhabdomyosarcoma	57	7.5
Endodermal sinus tumor	25	3.3
Primitive neuroectodermal sinus tumor	21	2.7
Germ cell tumor	17	2.2
Osteosarcoma	4	0.5
Ewing's sarcoma	6	0.8
Others	117	15.3
Mild acute malnutrition	140	19.2
Moderate acute malnutrition	41	5.6
Severe acute malnutrition	14	1.9
Overweight/Obese	38	5.2
Tumor metastasis	112	14.7
Stage‐IV	74	9.7

*Note*: Data are presented as median (interquartile range) or *n* (%).

The median age of the undernourished group was younger than that of the control group (*p* < 0.001), and a higher percentage of undernourished patients were male (*p* = 0.034). The proportion with malnutrition in neuroblastoma and hepatoblastoma patients was higher than that in patients with other diseases, but there was no significant difference. The nutritional characteristics of children with solid tumors at diagnosis are shown in Table 2.

**TABLE 2:** Nutritional status of children with solid tumors at diagnosis.

	Undernutrition (Z ≤ −1)	Control group (*Z* > ‐1)	*p*
Age (years)	3.50 (2.08,5.33)	2.16 (1.00,4.08)	<0.001
Gender (*n*, %)			0.034
Male	111(56.9)	257 (48.0)	
Female	84(43.1)	278 (52.0)	
Diagnosis			0.101
Neuroblastoma	88(28.3)	223 (71.7)	
Hepatoblastoma	12(17.9)	55 (82.1)	
Wilms tumor	32(24.4)	99 (75.6)	
Rhabdomyosarcoma	10(18.2)	45 (81.8)	
Others	53(31.9)	113 (68.1)	
Tumor metastasis	37 (19.0)	69 (12.9)	0.039
Stage‐IV	26 (13.3)	45 (8.4)	0.047

*Note*: Data are presented as median (interquartile range) or n (%).

### 3.2 Changes in nutritional status

#### 3.2.1 0–6 months

Using weight for age and BMI Z values to categorize nutritional status, 26.71% (*n* = 195) of the patients were undernourished (19.2% had mild acute malnutrition, 5.6% had MAM, 1.9% had SAM), and 5.21% (*n* = 38) were overweight or obese at diagnosis. After 3 months, the proportion of undernutrition slightly decreased by 25.86% (*n* = 188), and the proportion with overweight and obesity decreased by 2.89% (*n* = 21). After 6 months, the proportion of overweight and obese individuals decreased to 2.28% (*n* = 13), but the proportion of undernourished individuals increased by 29.77%.

**FIGURE 2:**
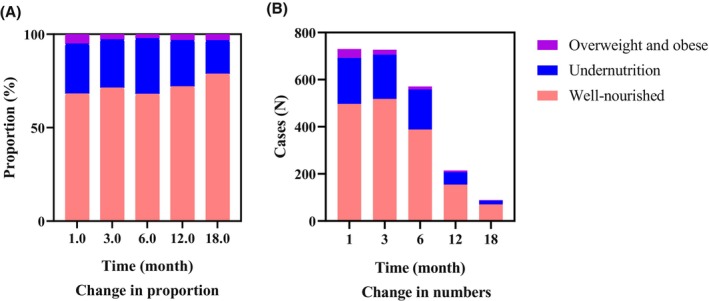
Change in nutritional status 0–18 months after diagnosis.

The changes in the BMI *Z* values continued during the stable treatment intensity period from 7 to 18 months (Figure 2B). One year after diagnosis, 24.77% (*n* = 53) of the patients were undernourished, and 3.27% (n = 7) were overweight or obese. After 18 months of treatment, the number of patients followed up decreased to 89, and 17.98% and 3.37% were still undernourished and overweight or obese, respectively (Figure 2A).

**FIGURE 3:**
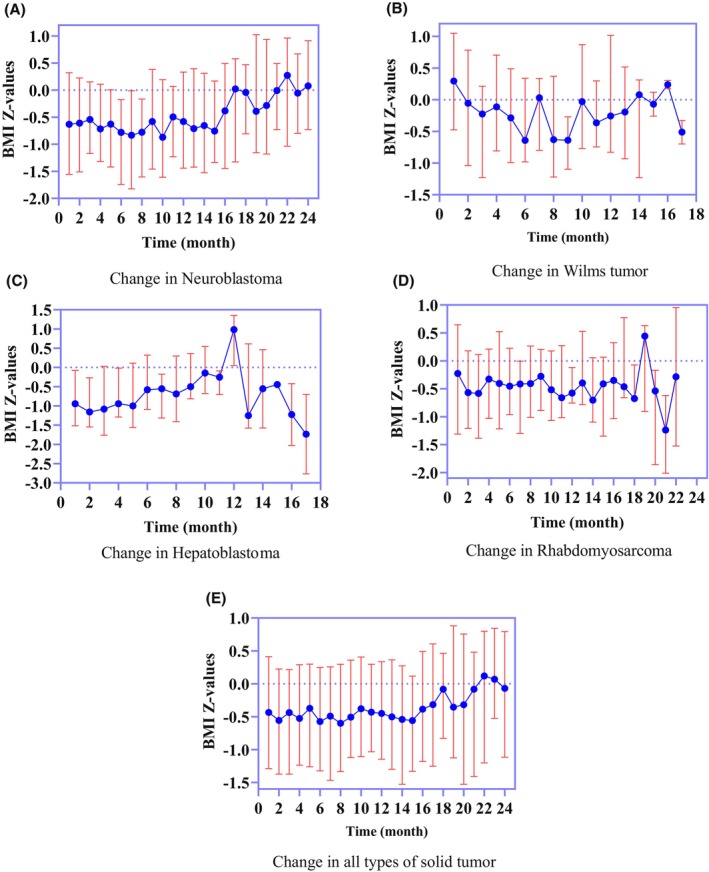
Nutritional status change in different types of solid tumor after diagnosis expressed in BMI *Z*‐values. Data are presented of patients with Neuroblastoma (A), Wilms tumor (B), Hepatoblastoma (C), Rhabdomyosarcoma (D), and all subset of solid tumors (E).

Although the BMI *Z* values of patients with solid tumors fluctuated to some extent, the change was not significant. The BMI *Z* values of children during the first 8 months after diagnosis changed irregularly (Figure 3E). The nutritional status of children decreased gradually from −0.38 (−1.11, 0.41) to −0.56 (−1.33, 0.12) in the 6 months beginning 10 months after diagnosis. Subsequently, the BMI *Z* values increased again to −0.08 (−0.83, 0.46) at month 18. Afterward, they tended to first decrease and then increase (Table S1), with the highest BMI *Z* value reaching 0.11 (−1.20, 0.80).

#### 3.2.2 Changes in BMI *Z* values of patients with different diagnoses

The variation in BMI *Z* values was different among the different types of solid tumors. Compared with patients with other malignant solid tumors, lower BMI *Z* values at diagnosis (−0.94 (−1.52, −0.07) were observed in patients with hepatoblastoma, followed by those with neuroblastoma (−0.63 (−1.56, 0.32)) and rhabdomyosarcoma (−0.22 (−1.31, 0.64)). The highest BMI *Z* values at diagnosis were in children with Wilms tumors (0.30 (−0.47, 1.05)). However, after 6 months, the nutritional status of children with neuroblastoma was worse than that of children with other solid tumors, with the lowest BMI *Z* value of −0.77 (−1.74, −0.17), and the most obvious reduction in malnutrition occurred in children with Wilms tumors (−0.64 (−0.98, 0.34).

In terms of the overall disease course, the nutritional status of children with rhabdomyosarcoma and neuroblastoma did not change significantly in the first 8 months (Figure 3A,D). Neuroblastoma patients showed an increase in their BMI Z values after 16 months, whereas rhabdomyosarcoma patients showed a significant change after 18 months. Children with hepatoblastoma showed a gradual improvement in nutritional status during the first 12 months of the disease course, a sudden sharp decline after 1 year, and then a steady state of nutritional risk. The Wilms tumor patients' BMI Z values deteriorated after diagnosis, increasing first and then decreasing 6 months later, with a large standard deviation due to the small sample size (Figure 3B).

### 3.3 Effects of malnutrition at admission on clinical outcomes

Poor nutritional status was associated with clinical outcomes as shown in Table 3. Patients with undernutrition status had a longer median length of the first hospitalization after diagnosis (15 (10, 21) vs. 12 (9, 17), *P* < 0.001). The total hospitalization cost (0.033 (0.027, 0.045) million yuan vs. 0.029 (0.018, 0.039) million yuan, *p* < 0.001) and antibiotic costs (925.2 (68.1, 2414.7) yuan vs. 403.4 (0, 1610.7) yuan, *p* < 0.001) were significantly higher in the undernutrition group than in the control group.

**TABLE 3:** Relationship between nutritional status and clinical outcome.

Outcomes	Undernutrition	Control group	*p* Value	Univariate OR (95% CI)	Multivariate OR (95% CI)
Length of stay (day)	15 (10,21)	12 (9, 17)	<0.001		
Hospitalization costs (million yuan)	0.033 (0.027, 0.045)	0.029 (0.018, 0.039)	<0.001		
Antibiotic costs (yuan)	925.18 (68.12, 2414.72)	403.40 (0, 1610.70)	<0.001		
ICU transfers	48 (24.9)	95 (18.1)	0.044	1.498 (1.010–2.224)	1.556 (1.008–2.402) *p* = 0.046
ICU duration (day)	1.89 (1.00,2.97)	1.91 (1.00, 2.10)	0.804		
Infection	64 (64.0)	129 (56.1)	0.858	1.392 (0.858–2.259)	1.265 (0.728–2.196) *p* = 0.405
Neutropenia	10 (10.0)	7 (3.0)	0.009	3.540 (1.307–9.587)	4.780 (1.571–14.553) *p* = 0.006
Parenteral nutrition (PN)	14 (7.2)	14 (2.6)	0.005	2.878 (1.346–6.154)	3.596 (1.453–8.898) *p* = 0.006
PN duration (day)	1.94 (0.18, 8.27)	1.97 (0.14, 4.23)	0.981		

*Note*: Data are presented as median (interquartile range) or *n* (%). The logistic regression model was adjusted for age, sex, diagnosis, whether undergone surgery, tumor metastasis, and the severity of the disease.

Children in the undernutrition group were more likely to be transferred to the ICU (univariate OR = 1.498 (95% CI: 1.010–2.224), *p* = 0.044), and the neutropenia risk was higher during hospitalization, with a higher proportion of PN‐receiving patients (univariate OR = 2.878 (95% CI: 1.346–6.154), *p* = 0.005). After adjusting for age, sex, diagnosis, surgery during hospitalization, severity of the disease and tumor metastasis, there was a significant correlation between nutritional status and the number of ICU transfers (multivariate OR = 1.265 (95% CI: 1.008–2.402), *p* = 0.046). Undernourished patients were more prone to neutropenia (multivariate OR = 4.781 (95% CI: 1.571–14.553), *p* = 0.006) and access to PN (multivariate OR = 3.596 (95% CI: 1.453–8.898), *p* = 0.006). Although there was a suggestion of a higher possibility of infection and a longer PN duration with undernutrition, there were no statistically significant differences between the two groups (Table 3).

## 4 DISCUSSION

Malnutrition is a common problem in pediatric patients with cancer. However, the reported prevalence and malnutrition criteria vary widely across all types of cancer.^6^, ^7^, ^23^ In this single center, we evaluated nutritional status at diagnosis and its changes in children with solid tumors as well as the correlation between malnutrition and short‐term clinical outcomes. The most common solid tumors in this study were neuroblastoma and hepatoblastoma, different from other studies.^24^

The prevalence of undernutrition varies from 8% to 23%, and the prevalence of overweight varies from 5% to 20%.^6^, ^25^ Our study found that the prevalence of malnutrition at diagnosis in children with solid tumors reached 26.71%, higher than the malnutrition rate reported by Karina^23^ and lower than that in patients with cancer in India (38%).^26^ In fact, different assessment criteria result in different malnutrition rates. Deniz Sul evaluated the nutritional status of the same group of children with cancer with different assessment tools. The prevalence of malnutrition according to weight for age, BMI, mid‐upper arm circumference (MUAC), and triceps skin‐fold thickness (TSFT) *Z* scores were 14.8%, 23.5%, 27.2%, and 21%, respectively.^19^ Patricia reported that the prevalence of different nutritional statuses at diagnosis, calculated according to the *Z* scores for H/A and BMI/A, was 29.3% malnourished, 49.5% adequate, and 21.2% overweight/obese in pediatric patients with solid tumors.^24^ In Central America, the moderate nutritional depletion proportion reached 18% by arm anthropometry; 45% of patients were severely depleted, and the proportion rose to 59% if serum albumin was added to the assessment criteria.^27^

All clinical methods for assessing nutritional status have limitations, particularly in children with cancer, as there is currently no clinical “gold standard.” Our center used height, weight, calculated BMI and their corresponding *Z* values to assess nutritional status. Although BMI is a valid indicator of nutritional status in children and adolescents across a wide range of ages (2–19 years),^28^ virtually all weight‐dependent measurements, such as weight and BMI, of nutritional status are problematic for children with cancer because 10% or more of their body weight may consist of tumors.^27^ Unfortunately, we only measured the weight and height of patients to calculate the dose of drugs in our center before this study, and the data were sometimes not even recorded in the electronic medical records in time. This hinders the assessment of children's nutritional status and the ability to provide more precise nutritional interventions. Therefore, we need to strengthen nutrition interventions and explore a variety of tools to better assess nutritional status, such as measuring the middle arm circumference and triceps skin‐fold thickness and using ultrasound tools to measure the thickness of the muscle; since research demonstrates that arm anthropometry is a more sensitive indicator of malnutrition.^27^, ^29^

Malnutrition in pediatric cancer is a complex, dynamic and multifactorial process. Zimmermann et al. studied malnutrition status in pediatric cancer patients from three tertiary care centers in Switzerland and showed that 5.8% of pediatric patients newly diagnosed with cancer were malnourished. During anticancer therapy, the cumulative incidence of malnutrition rose to 22% (70 patients) after 30 days, 36% (116 patients) after 60 days, and finally 47% (155 patients). The ratio of malnutrition time to total treatment time varied from 0% to 100% (median, 0; IQR, 0–22). During therapy, the cumulative incidence of malnutrition increased rapidly to nearly 50%.^30^ In this study, 26.71% of children were classified as undernourished and 5.21% as overweight and obese at the time of diagnosis. Six months after diagnosis, all of the children had decreased BMI *Z* scores, which then increased after 12 months. Similar to a prospective cohort study from a pediatric oncology department in the Netherlands, the number of moderate‐to‐severe malnutrition cancer patients decreased from 8.3% to 4.1% at 3 months, and the number of over‐nourished patients (BMI >2 SDS) increased from 4.5% to 6.6%.^31^

Malnourished children are more prone to discontinue treatment, and their event‐free survival rates are lower than those of other children.^27^ Cancer patients with malnutrition had a higher frequency of infection complications in the first 3 months after diagnosis, and the survival rate of malnourished children at 6 months was significantly lower than that of well‐nourished children.^32^ The association between malnutrition and clinical outcomes is also unclear, with some researchers suggesting that malnutrition is associated with worse outcomes,^33^ such as inferior tumor necrosis,^34^ increased risk of wound infection or sloughing and postoperative wound complications^35^; others assert that the link does not exist.^36^ Our study found that patients with undernutrition have an increased risk of adverse short‐term clinical outcomes, such as longer hospital stays, infections and ICU transfers. However, due to the lack of relevant data, the impact on long‐term clinical outcomes such as survival time, relapse and quality of life of children cannot be determined, representing a major limitation of our study. There is a window of opportunity to fully recognize the mechanistic changes occurring beneath the surface, and the long‐term effects of nutritional status and nutritional support on clinical outcomes are priorities for future study.

Scientific and reliable nutritional guidance and dietary principles are very important for patients with solid tumors, but in reality, patients' families often receive dietary guidance through nonprofessional channels or obtain no nutritional treatment consultation at all, causing these families to avoid some foods that are important sources of macronutrients and micronutrients. Mullee et al. conducted a multicenter observational study in seven hospital‐based oncology services in Ireland and found that 69% of patients obtained nutrition advice from professional and nonprofessional sources, mostly from family and friends, before their dietetic visits. Although this advice was rarely evidence‐based, frequently contradictory, and not clearly shown to be reliable, most patients followed the advice.^37^ It has been found that although many families of children with solid tumors believe that nutrition is essential, especially if diet‐related problems such as weight loss, muscle loss, nausea, vomiting, and loss of appetite are experienced, less than half or even fewer of these patients have access to a professional dietitian,^38^ as oncology dietetic services in healthcare systems are severely under‐resourced, and the registered dietitian nutritionist staffing in cancer centers is inadequate.^39^ There is an urgent need to strengthen awareness of the importance of standardized nutritional diagnosis and treatment of solid tumor patients and provide comprehensive nutrition screening, timely nutrition outpatient referral planning and individualized scientific nutrition guidance for such patients and their families.

Early nutritional counseling and dietary guidance can increase a patient's nutritional intake.^40^ Protein and energy‐dense oral nutritional supplements are effective in preventing weight loss in malnourished children.^32^ Brinksma et al. reported that 45.1% of cancer patients received nasogastric tube feeding for several days or weeks at any given time during the first year after diagnosis.^31^ In our center's clinical practice, the rate of tube feeding is much lower, as many families refuse it because they think it would be too painful for the child, although we do not have detailed data.

Our study was the most considerable research to date on the association between nutrition and solid tumor outcomes and the first to provide findings on the characteristics and nutritional status of children with malignant solid tumors in China. However, there are several limitations in this study. First, the study was based on retrospective data, and we could not obtain all anthropometric data if they had not been recorded. This is especially true for MUAC, which is a sensitive measure of nutritional status in children with cancer. Second, we were unable to access patient mortality data for our analysis. In our study, the patients were still alive at the last discharge. However, subsequent information about patients may have been lost. Last, the results from a growing number of studies have suggested that obesity can also be considered a significant problem affecting clinical outcomes,^41^, ^42^ and it is often a manifestation of poverty.^43^ In the context of this study, only a small number of children were overweight or obese, so we did not conduct further analysis of the impact of obesity on clinical outcomes. All these questions should be kept in mind in future studies. Future large‐scale cohort studies and assessments are needed to address these important questions and confirm our findings.

## 5 CONCLUSION

Our study shows that there is considerable malnutrition, especially undernutrition, in children with solid tumors. The variation in BMI *Z* values is different among different types of solid tumors by subset. Undernutrition can increase the length of stay, total hospitalization costs, antibiotic costs and the risk of ICU transfers and neutropenia. A better understanding of the role of nutritional status in pediatric solid tumors, early standardized patient nutrition assessment and targeted whole‐course nutrition intervention are greatly needed and can reduce the risk of adverse clinical outcomes, improve the quality of life of children, and economize iatrical resources.

## AUTHOR CONTRIBUTIONS

**Yongzhen Li:** Conceptualization (equal); data curation (equal); formal analysis (equal); methodology (equal); writing – original draft (lead); writing – review and editing (lead). **Zhongying Lu:** Data curation (equal); validation (equal). **Min Wu:** Data curation (equal); formal analysis (equal). **Ao Ma:** Data curation (equal); supervision (equal). **Wei Yao:** Data curation (equal); methodology (equal); supervision (equal). **Rui Dong:** Data curation (equal); supervision (equal). **Kai Li:** Methodology (equal); supervision (equal). **Kuiran Dong:** Conceptualization (equal); methodology (equal); supervision (equal). **Tian Qian:** Conceptualization (equal); methodology (equal); supervision (equal); writing – review and editing (equal).

## CONFLICT OF INTEREST STATEMENT

The authors report no conflicts of interest.

## Supporting information


Table S1.

## Data Availability

The data that support the findings of this study are available on request from the corresponding author. The data are not publicly available due to privacy or ethical restrictions.
