# Supplementary material for: Nutritional status associated with clinical outcomes in children with solid tumors: A retrospective cohort study from China
Source: Cancer Med. 2023 Dec 18;13(1):e6798. doi: 10.1002/cam4.6798 (PMC10807599; doi:10.1002/cam4.6798)
Supplement: Supplementary file 1 — Table S1. [file CAM4-13-e6798-s001.docx]

**Supplementary materials**

**Manuscript title:** Nutritional status associated with clinical outcomes in children with solid tumors: A retrospective cohort study from China

**Authors:** Yongzhen Li MS, Zhongying Lu BS, Ao Ma BS, Wei Yao PhD, Kai Li PhD, Rui Dong PhD, Min Wu BS, Kuiran Dong PhD, Tian Qian PhD

| **Supplementary Table 1.** Change in BMI Z-values of patients with solid tumors | | | |
| --- | --- | --- | --- |
| Time (month) | Median | P_25_ | P_75_ |
| 1 | -0.435 | -1.289 | 0.4123 |
| 2 | -0.557 | -1.374 | 0.2258 |
| 3 | -0.438 | -1.373 | 0.2142 |
| 4 | -0.525 | -1.237 | 0.2904 |
| 5 | -0.373 | -1.260 | 0.2987 |
| 6 | -0.574 | -1.324 | 0.2480 |
| 7 | -0.491 | -1.465 | 0.2581 |
| 8 | -0.600 | -1.335 | 0.2968 |
| 9 | -0.506 | -1.122 | 0.3600 |
| 10 | -0.379 | -1.105 | 0.4067 |
| 11 | -0.431 | -1.033 | 0.2959 |
| 12 | -0.450 | -1.146 | 0.3348 |
| 13 | -0.502 | -1.300 | 0.3637 |
| 14 | -0.540 | -1.527 | 0.2741 |
| 15 | -0.558 | -1.331 | 0.1179 |
| 16 | -0.385 | -1.179 | 0.4914 |
| 17 | -0.317 | -1.251 | 0.6075 |
| 18 | -0.081 | -0.829 | 0.4640 |
| 19 | -0.355 | -1.126 | 0.8813 |
| 20 | -0.317 | -1.527 | 0.7559 |
| 21 | -0.082 | -1.407 | 0.4804 |
| 22 | 0.1192 | -1.202 | 0.8001 |
| 23 | 0.0693 | -0.525 | 0.8437 |
| 24 | -0.069 | -1.116 | 0.7922 |

**Supplementary Figure 1** Flowchart of selecting study participants

2006 children with solid tumors in the oncology department at Children’s Hospital of Fudan University from January 2016 to December 2021

152 patients who did not readmission or follow-up less than 3 months

764 patients with solid tumors were included in final analysis

40 patients missing data of height, weight and biochemical examination

1050 pediatric solid tumors patients were diagnosis before January 2016

1854 patients collected during follow-up between 2016 to 2021
